# The effects of different rehabilitation training modalities on isokinetic muscle function and male athletes’ psychological status after anterior cruciate ligament reconstructions

**DOI:** 10.1186/s13102-023-00645-z

**Published:** 2023-03-27

**Authors:** Sofien Kasmi, Dorsaf Sariati, Raouf Hammami, Cain C. T. Clark, Mokhtar Chtara, Amri Hammami, Fatma Zohra Ben Salah, Ayoub Saeidi, Omar Ben Ounis, Urs Granacher, Hassane Zouhal

**Affiliations:** 1 Tunisian Research Laboratory ‘‘Sport Performance Optimization’’, National Center of Medicine and Science in Sports, Tunis, Tunisia; 2 Department of Physiotherapy, Posturology and Functional Rehabilitation, National Center of Medicine and Science in Sports, Tunis, Tunisia; 3 Higher Institute of Sport and Physical Education of Ksar Saïd, Tunis, Tunisia; 4 Research Laboratory: “Education, Motricity, Sports and Health” (UR 15JS01), Higher Institute of Sport and Physical Education of Sfax, University of Sfax, Sfax, Tunisia; 5 Centre for Intelligent Healthcare, Coventry University, Coventry, UK; 6 Laboratory of Physiology, Faculty of Medicine Ibn Jazar, Sousse, Tunisia; 7 Department of Physical Medicine and rehabilitation, Institute of Orthopedy M.T Kassab, La Manouba, Tunisia; 8 Department of Physical Education and Sport Sciences, Faculty of Humanities and Social Sciences, University of Kurdistan, Sanandaj, Kurdistan Iran; 9 Department of Sport and Sport Science, Exercise and Human Movement Science, University of Freiburg, Freiburg, Germany; 10 M2S (Movement Sport Science Laboratory), Univ. Rennes, Rennes, France; 11 Institut International des Sciences du Sport (2I2S), 35850 Irodouer, France; 12 Research Laboratory: Education, Motor Skills, Sports and Health (LR19JS01), Higher Institute of Sport and Physical Education of Sfax, University of Sfax, Sfax, Tunisia

**Keywords:** Injuries, Kinesiophobia, Neuromuscular training, Athletic performance, Muscle strength

## Abstract

**Background:**

Previously, researchers reported performance enhancements following long-term plyometric training in athletes with anterior cruciate ligament reconstruction (LCA). However, the effects of combined eccentric and plyometric training on measures of isokinetic strength and psychological statues in male athletes have not been examined yet. Knowledge on the effects of combined eccentric and plyometric training help to better plan and program rehabilitations sessions and thus return-to-sports.

**Objective:**

This study sought to compare the effects of three different rehabilitation training programs, eccentric training (ECC), plyometric training (PLYO), or combined eccentric and plyometric training (COMB), on psychological measures (kinesiophobia [TSK-CF], functional knee assessment, knee injury and osteoarthritis outcome score [KOOS], international knee documentation committee 2000 questionnaire [IKDC], and knee flexor and extensor isokinetic muscle performance (peak torque [PT], total work, ratio [R-HQ], and ratio of total work [R-TW]) at different angular velocities post ACL surgery in male elite athletes.

**Methods:**

Forty elite male athletes from different sports (e.g., athletics, team sports) with ACL reconstruction participated in this study. The study started after a 14-weeks post-surgery rehabilitation program, which was identical for all subjects. After this initial rehabilitation period, athletes were randomly assigned to three experimental groups, ECC (n = 10), PLYO (n = 10), and COMB (n = 10), and a control group (CON: n = 10). Testing was conducted pre- and post-the 6-weeks intervention period and included the TSK-CF, KOOS, and IKDC. Peak torque of the knee extensors/flexors was tested at 90, 180, 240 °/s, after the 6-weeks training program only.

**Results:**

Participants’ adherence rate was 100% across all groups and none reported any training or test-related injury. No significant between-group baseline differences (pre-6-weeks intervention) were observed for any of the reported psychological and muscle strength parameters. Significant group-by-time interactions were found for TSK-CF (p = 0.001, d = 2.85), KOOS (p = 0.001, d = 1.31), and IKDC (p = 0.001, d = 1.07). The post-hoc analyses indicated that COMB showed larger pre-post improvements for all psychological variables (p < 0.001, d = 2.95 to 13.15), compared with PLYO, ECC, and CON. Contrast analyses demonstrated that COMB yielded significantly greater improvements compared with CON, PLYO, ECC for all isokinetic parameters at all three angular velocities (all p < 0.001, d = 0.99 to 4.61).

**Conclusion:**

The results showed that COMB induced greater gains for measures of psychological status and isokinetic muscle strength compared with single-mode PLYO and ECC in elite male athletes during a post-surgery ACL rehabilitation period. Accordingly, it is recommended to implement COMB as an effective rehabilitation means to improve knee function in male elite athletes.

**Trial registration:**

This study does not report results related to health care interventions using human participants and therefore it was not prospectively registered.

## Background

An anterior cruciate ligament (ACL) rupture is unfortunately a common injury in sports, particularly in team sports, that is often caused when performing cutting, jumping, and/or pivoting actions [1]. A recent systematic review including 45 studies with male and female athletes from 13 team ball sports revealed an incidence rate of 0.05 (95% CI 0.03–0.07) per 1000 player-hours of non-contact ACL injuries in male athletes [1]. Incidence rates were higher during competition (0.48 per 1000 player-hours, 95% CI 0.32–0.72) compared with training (0.04 per 1000 player-hours, 95% CI 0.02–0.07).

For an athlete, the ultimate goal after ACL reconstruction (ACLR) and rehabilitation is to regain normal range of motion, knee joint stability, sufficient muscle strength and power, psychological well-being [2–5], and to avoid re-injury [6]. Although surgery restores mechanical knee stability [7], athletes are often affected by psychosocial factors such as fear of reinjury [2, 5, 8–10]. In addition, neuromuscular factors such as a considerable loss of muscle strength and mass, and inhibited quadriceps activation may have a negative impact on the post-operative period [7–9, 11–13]. Therefore, adequate and effective intervention programs are needed that have the potential to restore knee function. However, there is still controversy regarding the most effective training modalities that practitioners should prescribe after the rehabilitation period.

Plyometric jump training could be a candidate to promote the return-to-sports process after ACL reconstruction by improving quadriceps muscle strength and knee function. However, the intervention has not yet been examined in male athletes during this critical rehabilitation period [8, 14]. Chmielewski et al. [8] suggested that both, low and high intensity-plyometric training have the potential to induce positive changes in knee isokinetic muscle strength, and psychosocial well-being, which would support the return-to-sports process after ACL reconstruction. Kasmi et al. [5] reported for female athletes with ACL reconstruction (ACLR) that the combination of plyometric and eccentric strength training is more effective than single-mode plyometric or eccentric training on both, stability and functional performance.

The same authors postulated that combined plyometric and eccentric training appear to be particularly effective to improve measures of dynamic balance, muscle strength and power, and psychological well-being (e.g., knee injury and osteoarthritis outcome score [KOOS], Tampa kinesiophobia score [TSK-CF]) compared with single-mode plyometric training in female athletes following ACL reconstruction. However, there is no study available, solitarily in male athletes, that has examined the effects of combined plyometric and eccentric training versus single-mode eccentric or plyometric training on isokinetic muscle strength (i.e., peak torque, power and ratio hamstring/quadriceps) and athletes’ psychological status (i.e., kinesiophobia [TSK-CF], functional knee assessment, knee injury and osteoarthritis outcome score [KOOS], international knee documentation committee 2000 [IKDC]). Evidence from female athletes [5] showed that combined plyometric and eccentric strength training has greater effects on muscle strength and athletes’ psychological status after ACL reconstruction. However, hardly any information is available in the literature regarding the effects of combined training (e.g., eccentric and plyometric training) in contrast to single-mode plyometric or eccentric training on muscle strength, psychological status, and knee function in male athletes after ACL reconstruction.

Therefore, the purpose of this study was to examine the effects of 6-weeks of combined eccentric-plyometric training versus single-mode eccentric and versus single-mode plyometric training during ACLR rehabilitation of elite male athletes from different sports (e.g., athletics, judo, team sports). We analyzed psychological measures (TSK-CF), functional knee assessment, (KOOS), (IKDC), and knee flexor and extensor isokinetic muscle strength (i.e., limb symmetry index [LSI], peak torque [PT], total work, hamstring-quadriceps ratio [R-HQ], and ratio of total work [R-TW]) at different angular velocities of athletes’ post-surgery of ACLR in elite athletes.

Based on relevant literature data and similar to the previous studies conducted with female athletes [2–5, 8, 9, 12–14], we hypothesized that combined plyometric and eccentric strength training would lead to greater improvements in measures of psychological kinesiophobia status, functional knee assessment and isokinetic muscle strength performance compared with single-mode plyometric or single-mode eccentric training in male athletes with ACLR.

## Methods

The current study with male athlete’s complements findings from a previous study [5] with female athletes from our research group. The novelty of this study is that we examined the exercise effects in male athletes and that we examined additional outcome parameters such as KOOS, IKDC, LSI, PT, TW, R-HQ, R-TW at different angular velocities, and athletes’ psychological (TSK-CF).

**Fig. 1:**
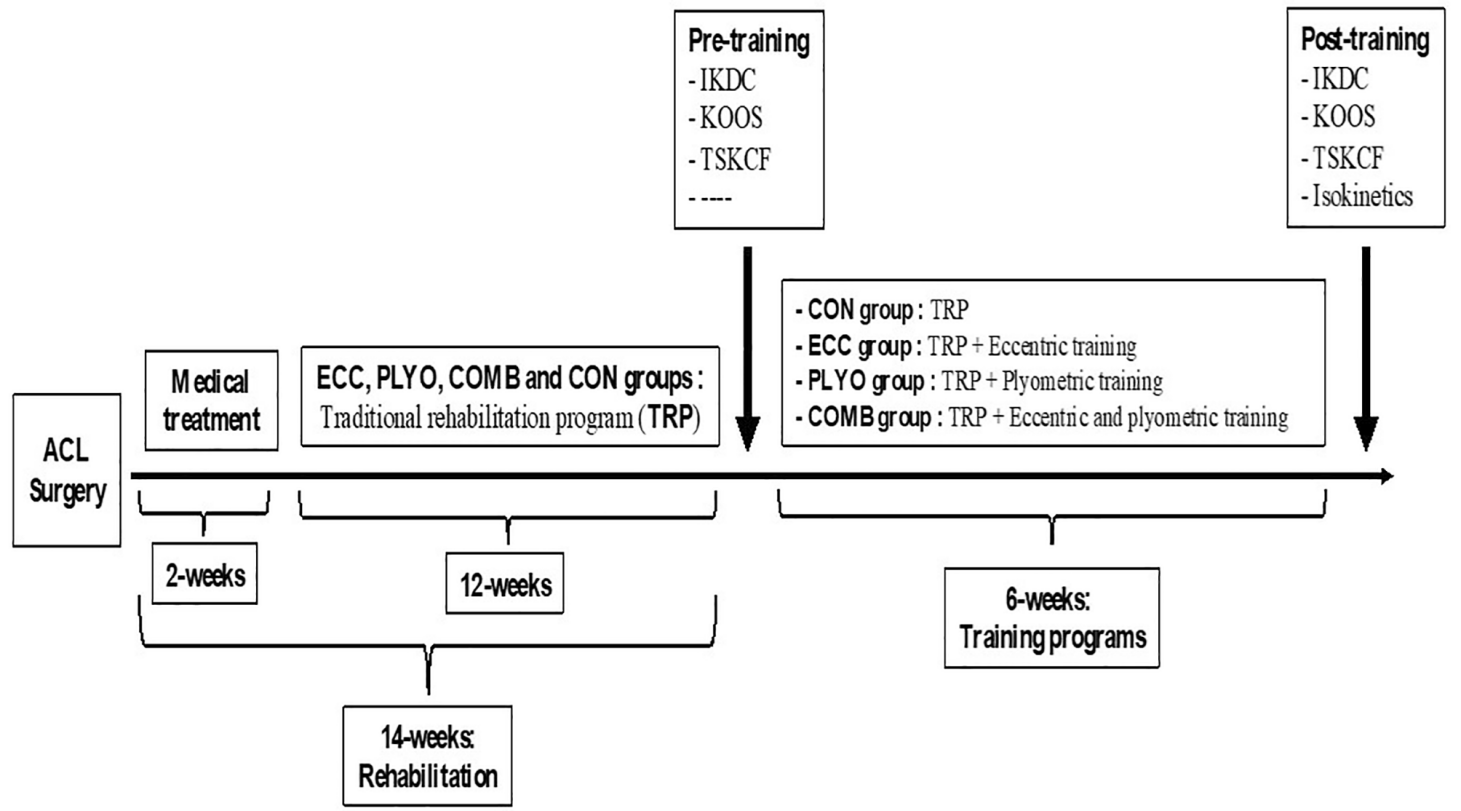
Experimental protocol adopted in the present study

Two weeks after ACL-surgery, all participating athletes completed a 12-weeks standardized rehabilitation program. After this period, the 6-weeks experimental period started (Fig. 1). The three experimental groups (n = 10; for each group) were: 1) single-mode eccentric group (ECC), single-mode plyometric group (PLYO), and 3) combined eccentric and plyometric group (COMB).

The control group (CON, n = 10) was recruited from the same cohort of elite athletes who had undergone an ACL reconstruction. Athletes in CON were tested and instructed to follow their traditional rehabilitation program consisting of flexibility, balance (i.e., bibedal and unipedal stance) and submaximal runing exercises [15].

Testing was conducted pre-and post-the 6-weeks intervention period and included the assessment of functional knee assessment (IKDC and KOOS) and psychological kinesiophobie status (TSKCF). However, isokinetic parameters were assessed just after the 6-weeks training program.

## Participants

Forty elite male athletes, who suffered an ACL injury and received rehabilitation volunteered to participate in this study (Table 1).

**Table 1:** Baseline characteristics of the participants 14-weeks rehabilitation after ACL-reconstruction.

Variables	CON (n = 10)	ECC (n = 10)	PLYO (n = 10)	COMB (n = 10)
Sport: T/I	8/2	8/2	8/2	8/2
Training session/week (n)	7	7	7	7
Dominant Limb: R/L (n)	7/3	8/2	7/3	5/5
Operated Limb: R/L (n)	6/4	8/2	8/2	8/2
Age (year)	20.4 ± 3.34	20.30 ± 2.83	20.30 ± 2.54	20.60 ± 3.80
Body mass (kg)	75.50 ± 5.23	78.00 ± 8.12	78.20 ± 9.33	79.50 ± 4.84
Body height (cm)	179.60 ± 4.74	180.40 ± 10.12	179.50 ± 8.13	184.00 ± 5.37
BMI	20.95 ± 1.09	21.59 ± 1.29	21.68 ± 1.65	21.56 ± 1.04

CON = control group; ECC = eccentric group; PLYO = plyometric group; COMB = combined group; F = Female; M = Male; L = Left; R = Right; T = Team sport; I = Individual sport; BMI = body mass index

**Study inclusion criteria were:**

- athletes who performed systematic sports on an international level and were members of the Tunisian national team in their respective sport (e.g., athletics, judo, team sports, etc.);
- athletes who practiced their sport for more than 10 years and exercised between 6 and 8 times per week including competition;
- athletes with a non-contact ACL injury received during the sporting activity (training and/or competition).
- Males’ athletes.

**Study exclusion criteria were:**

- athletes who received ACL surgery with techniques other than the bone patellar-tendon, bone graft (BPTB), or who were operated by different surgeons and rehabilitated by several physical therapists;
- athletes with a history of muscle or joint injuries;
- athletes with poor attendance rates (below 80%);
- athletes who had already followed a preoperative rehabilitation program;
- athletes with complications after surgery;
- pregnant athletes and those who used anti-inflammatory drugs or muscle relaxers.

Participants provided written informed consent after a thorough explanation of the study objectives, the procedures, risks, and benefits of the study.

The study was conducted according to the latest version of the Declaration of Helsinki and the protocol was fully approved by the Ethics Committee of the National Centre of Medicine and Science of Sports of Tunis (CNMSS-LR09SEP01) before the commencement of the assessments.

The required sample size was calculated with G*Power (Version 3.1, University of Dusseldorf, Germany) [16]. The a priori power analysis was computed with an assumed power of 0.95, an alpha level of 0.01, and an effect size of Cohen’s Cohen’s f = 0.53) for the IKDC scores [17]. The analysis revealed that a total sample size of N = 32 would be sufficient to detect a significant group-by-time interaction effect. Accordingly, 40 participants were enrolled to account for potential drop-outs due to injuries.”

## ACL-surgery and rehabilitation

All surgeries were performed at the Hospital Ortopédico (Allysa, Tunisia) by two knee surgeons who had at least 20 years of technical experience with ACL reconstruction. Two weeks after surgery and 12-weeks before the intervention period, all participants received the same rehabilitation protocol (phases 1, 2, 3, and 4) consisting of edema and inflammation control, expansion of the range of motion, improvement of stability, and strength training [15].

### Randomization

Participants were randomly allocated to three experimental groups and a control. Every participant received an ID number which was entered into an online randomization calculator (https://www.randomizer.org/) which allocated the participants into groups. Group allocation was accomplished by controlling for age, and BMI of the study participants. In addition, the order of each trial was changed randomly among participants, to avoid learning effects and fatigue. After 12 weeks of rehabilitation post ACL surgery, this latter procedure was performed by an independent researcher who was not involved during the rehabilitation process.

### Training programs

Participants of the ECC, PLYO, and the COMB groups performed two weekly training sessions over a 6-weeks intervention period with ~ 60 min per session and an overall number of 12 training sessions [5] in addition to the traditional program [15]. The training volume (training weeks, sets, repetitions, and duration) was equal between the intervention and the control groups.

Each training session started with a standardized 15-minute warm-up, including submaximal intensity running, dynamic stretching, calisthenics, and preparatory exercises (e.g., balance and landing, squatting, jumping exercises) at a progressively increased intensity. The rehabilitation program was performed for all groups at the National Medical Sports Center of Tunis, Tunisia. Training was delivered by expert strength and conditioning coaches from the National Medical Sports Center of Tunisia.

## Dependent variables

### Psychological status

#### The tampa kinesiophobia score (TSK-CF)

The TSK consists of 17 questions with a total score ranging from 17 to 68, where higher scores indicate greater fear of movement/injury [18]. Lundberg et al. MK Lundberg, J Styf, SGJPt Carlsson and practice [19] reported TSK scores for 2 groups of different patients, a median of 30 for the first group and 44 for the second group. Other studies suggest several different cut-off values, ranging from greater than 35 to greater than 44, which were used later [18–20]. In our study, we used the TSK scale as described by [21, 22], with a 17-item TSK that assesses fear of movement / (re) injury. Patients are rated for each item on a 4-point Likert scale, with alternatives ranging from “strongly disagree” to “strongly agree”. Items 4, 8, 12, and 16 are noted inversely. Total scores range from 17 to 68; higher scores reflect a state of fear of movement / (re) injury.

#### The KOOS score for LCA injury (knee injury and osteoarthritis outcome score)

The KOOS is a self-administered questionnaire that assesses the consequences of a knee injury in the short and long-term. It contains 42 items in 5 separately scored subscales; pain, other symptoms, function in daily living (ADL), function in sport and recreation (sport/recreation), and quality of life (QoL) related to the knee. Several randomized studies using KOOS have previously been published. For example, T Nau, P Lavoie and N Duval [23] compared two ACL reconstruction methods (KJ vs DIDT), they found significant differences between the 2 groups and at various postoperative times, in daily functional life, that of sport/leisure training and knee-related quality of life. For this reason, the score is calculated for each subscale, where 100 indicates the absence of any problem and 0 indicates extreme problems [24].

The five KOOS subscales are scored separately: Pain (nine items); Other Symptoms (seven items); Function in daily life (17 items); Function in Sport and Leisure (five items); Knee-Related Quality of Life (four items). A Likert scale was used, where all items had five possible response options marked from 0 (no problem) to 4 (extreme problems) and each of the five scores is calculated as the sum of the items included [25]. Scores are transformed onto a scale of 0 to 100, with zero representing extreme knee problems and 100 representing no knee problems at all as in the Common Orthopedic Rating Scales and Generic Measures. Scores between 0 and 100 represent the percentage of the total possible scores achieved [25].

#### The IKDC score (international knee documentation committee 2000)

The IKDC 2000 normative database can be used as a standard for positive patient-reported outcomes [26]. The original form contained 18 questions, in which total scores are expressed as a percentage from 0–100%, with higher scores representing better knee function and fewer symptoms. The IKDC 2000 is a reliable self-report with valid outcome measures [27], consisting of a 10-question rating converted to a 100-point scale with a 95% confidence interval. This form has been tested on a large number of patients, with various knee disorders, and is valid [27].

#### Isokinetic muscle strength performance assessment of the knee extensors and flexors

Isokinetic refers to a mode of dynamic voluntary muscle contraction using a constant speed with a self-adjusting resistance. This speed regulation is provided by an external device called an “isokinetic dynamometer” [28]. The tests were carried out using an isokinetic dynamometer (CybexNorm® (6000), Manufacturer, Ville, USA), which allowed the measurement of the knee flexor and extensor peak torque (PT), the total work (TW), ratio R-PT and R-TW.

The subject was placed in a seated position, with a posterior tilt of the trunk of 15° to 20° from the vertical line. Participants were restrained by straps at the trunk and thigh to avoid any compensation, and the hands were gripped to the seat by side handles. The recording took place over an angular sector of 90°, where the active extension corresponds to 0°, and the gravity correction was made at 45 ° [29].

Typical concentric H/Q ratios in healthy subjects range from 0.5 to 0.8, with a higher ratio at faster angular knee velocities during isokinetic testing [30–32]. It has been found that athletes with a concentric H/Q ratio closer to 1.0 may be associated with a reduced risk of hamstrings strain [32]. Also, a concentric H/Q ratio closer to 1.0 in athletes with ACL injury has been suggested to reduce the risk of an anterolateral subluxation of the tibia [33]. It has been shown that bilateral lower limb strength asymmetry (LSI) can be calculated in different ways: (1) injured / uninjured; (2) right / left; (3) stronger / weaker. The first method can only be applied to injured athletes [34]. The Limb Symmetry Index (LSI) is commonly used in isokinetic testing to quantify quadriceps and hamstring deficits between the involved limb (operated limb) and the healthy contralateral limb (no operated limb) [35]. An LSI ≥ 90% in a single test was considered normal [36, 37].

### Statistical analysis

Data were presented as group means values and standard deviations (SD). Normal distribution of data was assessed, and confirmed, using the Shapiro-Wilk test. One-way analysis of variance (ANOVA) with repeated measures was used to evaluate differences between groups in means differences (MD = operated – non operated legs) ratio peak torque and total work and to evaluate differences between groups in index LSI (ILSI = LSI peak torque or LSI total work − 100) peak torque and total work on knee extensors and flexors. Post-hoc comparisons were calculated using the Tukey-HSD test. The effect size (ESs) was calculated for all ANOVA using partial eta-squared (η2). The values of 0.01, 0.06, and 0.15 were considered small, medium, and large cut-off points, respectively [38, 39].

Moreover, differences between the operated and non-operated leg were computed using paired t-tests. Effect sizes (ES) were determined by converting partial eta-squared from the ANOVA output to Cohen’s d. ES can be classified as trivial (< 0.2), small (0.2–0.49), medium (0.5–0.79), or large (≥ 0.8) [38, 39]. For the varying groups, i.e., combined, plyometric, eccentric, and control, contrast analyses [40] was also be carried out to specifically test the following hypotheses; H1) COMB would yield greater improvements in the outcome measures than all other groups (PLYO, ECC, CON), H2) COMB will yield greater improvements in the outcome measures than PLYO and ECC, and H3) COMB will yield greater improvements in the outcome measures than CON. This approach yields a comparison of one (or more) condition(s) vs. the grand mean of the specified contrasts.

Indeed, post-hoc analyses, while useful, do not necessarily yield sufficient insight into multiple levels or detailing patterns in response; whereas, contrast analysis allows researchers to test theory-driven expectations directly against an empirically derived group or cell means [40]. Statistical analyses were performed using SPSS (SPSS Inc., Chicago, IL, version. 20.0), and significance was accepted, a priori at p < 0.05.

## Results

### Psychological measures

Analysis indicated a significant main effect of time (all, p < 0.001, d = 4.61 to 8.71, very large) for TSK-CF, KOOS, and IKDC. Significant group x time interactions were noted for TSK-CF (p = 0.001, d = 2.81), IKDC (p = 0.001, d = 1.06), and KOOS (p = 0.001, d = 1.3). Post hoc analysis indicated that the combined group showed significant improvements post-intervention (p < 0.001, d = large), to a greater extent than the plyometric, eccentric and control groups on all variables.

The combined group achieved significantly greater improvements in IKDC, TSK-CF, and KOOS (137.2, -52.2 and 80.6% respectively; d = large) than plyometric (114.7, -36.5 and 59.6% respectively), ECC (111.9, -28.7 and 60.9% respectively), and control (69.2%, -16.1 and 60.5% respectively) groups. In addition, the plyometric group improved to a significantly greater extent (p < 0.05) than eccentric and controle groups on IKDC and TSK-CF. Furthermore, the eccentric group improved significantly more (p < 0.05) than the controle group in all psychological measurements. Contrast analysis showed that combined training elicited greater improvements (all P < 0.001), (Table 2).

**Table 2:** Effects of the different 6-weeks rehabilitation training modalities on psychological status in male’s elite athletes

	Group	Pre	Post	Delta %	Cohen’s *d* (before vs. after) [95CI%]	ANOVA *p*-value (Cohen’s *d*)		
						Time	Group	Group x Time
**IKDC**	**CON (n = 10)**	44.40 ± 9.28	71.39 ± 1.92	60.08	2.91	0.001 (4.61)	0.001 (1.05)	0.001 (1.08)
	**ECC (n = 10)**	43.30 ± 10.48	87.15 ± 3.67	101.3^a^	4.19			
	**PLYO (n = 10)**	43.40 ± 8.18	80.71 ± 2.89	109.0^a^	5.78			
	**COMB (n = 10)**	43.90 ± 9.67	98.64 ± 1.42	124.7^a,b^	5.66			
**TSKCF**	**CON (n = 10)**	48.40 ± 1.84	41.10 ± 2.18	-15.1	-3.97	0.001 (8.10)	0.001 (2.61)	0.001 (3.30)
	**ECC (n = 10)**	50.00 ± 1.66	35.60 ± 1.58	-28.8^a^	-8.47			
	**PLYO (n = 10)**	50.10 ± 1.73	32.30 ± 1.25	-35.5^a^	-10.30			
	**COMB (n = 10)**	48.70 ± 2.00	23.00 ± 1.05	-52.8^a,b,c^	-12.83			
**KOOS**	**CON (n = 10)**	49.75 ± 2.52	79.82 ± 1.52	60.4	11.93	0.001 (9.48)	0.001 (1.69)	0.001 (1.39)
	**ECC( n = 10)**	52.54 ± 4.36	82.56 ± 1.22	57.1^a^	6.88			
	**PLYO (n = 10)**	52.04 ± 3.48	81.23 ± 0.42	56.1	8.39			
	**COMB (n = 10)**	52.40 ± 3.37	93.26 ± 1.22	78.0^a,b,c^	12.13			

CON = control group; ECC = eccentric group; PLYO = plyometric group; COMB = combined group

### LSI peak and ratio peak torque and total work

#### Post-hoc analyses

For ratio peak torque and total work at 90, 180, and 240°, the means differences (MD = operated –non-operated legs) are less important in combined than in eccentric, and plyometric groups (p < 0.001) (Table 4). In addition, the eccentric group showed best (p < 0.001) MD than the control and plyometric groups. Finally, the control group is the best from the plyometric group (p < 0.005) (Table 3).

**Table 3:** Effects of 6-weeks of combined eccentric and plyometric training versus single-mode plyometric and single-mode eccentric training versus control on the ratio peak torque and total work in male’s elite athletes

Angular velocity	Group	Ratio PT (N.Kg^− 1^)						Ratio TW				
		Op Leg	Non Op Leg	Delta %	ES	ANOVA *p*-value (Cohen’s *d*)		Op Leg	Non Op Leg	Delta %	ES	ANOVA *p*-value (Cohen’s *d*)
**90 °/s**	**CON (n = 10)**	81.73 ± 12.57	57.59 ± 2.34	-29.5 ^c^	1.92	**0.001** (0.09)		86.18 ± 9.13	61.17 ± 4.12	-29.00 ^c^	2.74	**0.001** (0.4)
	**ECC (n = 10)**	43.52 ± 4.54	57.59 ± 4.66	32.3 ^c^	3.10			48.48 ± 4.01	60.29 ± 6.42	24.4 ^a,c^	2.94	
	**PLYO (n = 10)**	94.10 ± 6.38	56.61 ± 2.96	-39.8	5.88			97.25 ± 9.81	59.49 ± 4.82	-38.8	3.85	
	**COMB (n = 10)**	56.50 ± 2.67	53.98 ± 1.64	-4.50 ^a,b,c^	0.94			59.81 ± 2.54	57.77 ± 1.55	-3.4 ^a,b,c^	0.80	
**180 °/s**	**CON (n = 10)**	83.85 ± 10.54	57.55 ± 3.48	-31.4 ^c^	2.49	**0.001** (0.03)		86.36 ± 7.30	61.89 ± 3.83	-28.3 ^c^	3.35	**0.001** (0.15)
	**ECC (n = 10)**	45.21 ± 2.51	58.75 ± 3.59	29.9 ^a,c^	5.40			45.97 ± 2.66	61.18 ± 4.73	33.1 ^c^	5.71	
	**PLYO (n = 10)**	97.47 ± 5.66	58.23 ± 3.40	-40.3	6.93			97.80 ± 8.85	61.01 ± 4.31	-37.6	4.16	
	**COMB (n = 10)**	56.56 ± 2.17	54.64 ± 1.86	-3.40^a,b,c^	0.89			59.81 ± 2.43	58.11 ± 1.64	-2.80 ^a,b,c^	0.70	
**240 °/s**	**CON (n = 10)**	79.12 ± 3.34	58.14 ± 3.81	-26.5 ^c^	6.29	**0.001** (0.05)		83.17 ± 4.76	61.62 ± 3.60	-25.9 ^c^	4.53	**0.001** (0.17)
	**ECC (n = 10)**	45.10 ± 2.07	58.50 ± 4.26	29.7 ^c^	6.47			45.47 ± 4.10	59.40 ± 3.86	30.6 ^c^	3.39	
	**PLYO (n = 10)**	98.97 ± 6.34	58.87 ± 4.86	-40.5	6.32			100.76 ± 6.46	61.15 ± 5.60	-39.3	6.13	
	**COMB (n = 10)**	57.01 ± 2.37	54.40 ± 1.70	-4.6 ^a,b,c^	1.10			59.94 ± 2.00	57.99 ± 1.84	-3.30 ^a,b,c^	0.98	

a = significantly different from CON; ^b^ = significantly different from ECC; ^c^ = significantly different from PLYO; (p < 0.01); Op Leg: operated leg, Non-Op Leg: non-operated leg, PT: pic torque, TW: total work

For LSI peak torque and total work at 90, 180, and 240° for extensors, the index LSI is less important in combined than in eccentric, plyometric, and control groups (p < 0.001) (Table 4). In addition, the eccentric group showed best (p < 0.001) ILSI than the plyometric and control groups (Table 4). For LSI peak torque at 90, 180, and 240° for flexors, the ILSI is less important in combined and plyometric groups than in eccentric and control groups (p < 0.001) (Table 4).

**Table 4:** Effects of 6-weeks of combined eccentric and plyometric training versus single-mode plyometric and single-mode eccentric training versus control on the limb symmetry index peak torque and total work of the knee extensors and flexors in male’s elite athletes

Angular velocity	Group	Extensors			Flexors	
		LSI-PT (Nm/kg)	LSI-TW (J)		LSI-PT (Nm/kg)	LSI-TW (J)
**90 °/s**	**CON (n = 10)**	67.40 ± 9.54	70.60 ± 10.06		77.10 ± 6.03	77.30 ± 6.72
	**ECC (n = 10)**	117.20 ± 18.89 ^a,c^	115.40 ± 16.53 ^a,c^		82.10 ± 12.29	84.10 ± 12.39
	**PLYO (n = 10)**	69.40 ± 4.70	72.00 ± 6.80		105.80 ± 6.46 ^a,b^	106.80 ± 5.01 ^a,b^
	**COMB (n = 10)**	98.70 ± 0.82 ^a,b,c^	98.40 ± 0.70 ^a,b,c^		102.30 ± 2.87 ^a,b^	101.40 ± 2.22 ^a,b^
**ANOVA.** ***p*** **-value**		**0.001**	**0.001**		**0.001**	**0.001**
**180 °/s**	**CON (n = 10)**	68.60 ± 6.06	71.30 ± 8.30		78.40 ± 4.74	79.40 ± 5.42
	**ECC (n = 10)**	110.80 ± 13.30 ^a,c^	114.20 ± 14.46 ^a,c^		78.60 ± 10.59	79.70 ± 12.29
	**PLYO (n = 10)**	70.60 ± 5.30	72.30 ± 4.92		105.70 ± 7.69 ^a,b^	106.20 ± 5.22 ^a,b^
	**COMB (n = 10)**	98.30 ± 0.82 ^a,b,c^	98.50 ± 0.85 ^a,b,c^		102.70 ± 2.79 ^a,b^	102.50 ± 2.42 ^a,b^
**ANOVA.** ***p*** **-value**		**0.001**	**0.001**		**0.001**	**0.001**
**240 °/s**	**CON (n = 10)**	68.60 ± 6.93	71.30 ± 7.83		79.70 ± 4.32	79.20 ± 5.63
	**ECC (n = 10)**	112.50 ± 13.63 ^a,c^	114.20 ± 14.77 ^a,c^		83.60 ± 13.33	83.40 ± 14.37
	**PLYO (n = 10)**	71.90 ± 5.04	73.50 ± 4.33		106.10 ± 6.23 ^a,b^	107.70 ± 6.17 ^a,b^
	**COMB (n = 10)**	98.40 ± 0.84^a,b,c^	98.60 ± 0.84 ^a,b,c^		103.00 ± 3.30 ^a,b^	103.10 ± 3.48 ^a,b^
**ANOVA** ***p*** **-value**		**0.001**	**0.001**		**0.001**	**0.001**

a = significantly different from CON; ^b^ = significantly different from ECC; ^c^ = significantly different from PLYO; (p < 0.01); PT: pic torque, TW: total work

For LSI total work at 180 and 240°, the ILSI is less important in COMB than in eccentric, plyometric, and control groups (p < 0.001) but in angular speed 90°, combined, and plyometric has an ILSI less important than eccentric and control groups (p < 0.001).

### Contrast analyses

Relative to contrast analyses, all specified hypotheses were tested and are detailed in Tables 3–4.

### Isokinetic strength

The LSI index was lower in combined than control group for all angular speeds (all p < 0.001). We found also that for index LSI, the deficit is less important in combined than in plyometric and eccentric (all p < 0.0001). Finally, the combined group yielded a significantly better LSI index in all speeds (all p < 0.001), (Table 3).

### For LSI peak torque and total work

The LSI index was lower in eccentric and plyometric than control group for all angular speeds (all p < 0.001). We found also that for the LSI index, the deficit was lower in combined than in plyometric and eccentric (all p < 0.0001). Finally, we found that combined yielded a significantly better LSI index in all speeds (all p < 0.0001), (Table 4).

## Discussion

The main findings of this study were that COMB yielded significantly greater improvements compared with CON and single-mode PLYO, ECC for the applied isokinetic parameters at the three angular velocities. Overall, changes after combined plyometric and eccentric exercise appear to facilitate a return-to-sports participation after ACL reconstruction.

Moreover, the results showed that combined training is more effective for psychological and functional self-reported parameters in knee rehabilitation after LCA reconstruction (we observed intensive increase of scores on KOOS, IKDC and TSK-CF questionaries’) compared with ECC, PLYO, and CON programs. With regards to prior reported training studies [3, 5, 9], the improved psychological and strength related components was more evident with COMB programs after ACL reconstruction. Overall, changes after combined plyometric and eccentric exercise appear to facilitate a return to sports participation after ACL reconstruction.

The present results showed an improvement in muscle strength performance, with the largest effects observed in the COMB group. These results supports the adaptive potential of muscle morphology in athletes after ACL reconstruction [3, 7]. After strength training, morphological changes occurs and, in addition to structural changes in the muscle itself, can include an increase in muscle fiber size and changes in aspects of fiber-type composition and connective tissue [3, 5]. Moreover, during both combining concentric and eccentric exercise, the greater mechanical tension exerted on muscle tissue promotes a larger exercise-induced muscle damage, which is one of the key mechanisms to muscle hypertrophy [7, 41].

Although traditional strength training is performed with the same external load during concentric and eccentric muscle actions, evidence suggests that eccentric overload exercises result in larger muscle strength and hypertrophy [42]. In addition, studies with athletes have demonstrated higher increments in muscle strength, mass, and athletic performance through eccentric training compared with traditional (concentric/eccentric) strength training [3]. Vidmar et al. [7] demonstrated that isokinetic eccentric, compared with constant load eccentric, training for the quadriceps muscle is more effective for measures of muscle mass (+ 17–23% vs. +5–9%), as well as for isometric (+ 34% vs. +20%) and eccentric (+ 85% vs. +23%) peak torques in thirty recreational male athletes (25 years old) with ACL reconstruction.

Hence, it can be speculated that the superiority of the combined group over the plyometric and the eccentric groups could, therefore, be explained by the fact that participants could benefit from training responses, due to the low baseline levels of strength and power. Hence, current recommendations support that rehabilitation after ACL reconstruction should be initiated as early as possible [43].

Furthermore, after LCA reconstruction, the execution of strength and plyometric training may be an important determinant of functional knee assessment in athletes [3, 8]. Indeed, plyometric exercise enhanced eccentric force production in the lower body to a greater degree than traditional resistance training, which predominantly favored concentric adaptations, and subsequently, may elicit a positive effect on functional knee assessment in general [44]. These positive effects occurring in response to eccentric training have been attributed to a beneficial modification of several factors of muscle morphology (cross-sectional area, fiber typing, pinnation angle and working range,), in addition to peripheral and central neural activity alpha motoneuron recruitment/firing, brain activation, corticospinal excitability and sarcolemma activity) [45]. Thus, by improving quadriceps muscle strength and knee function, COMB exercise might assist the return to sports participation after ACL reconstruction. Hence, it seems that the addition of strength and plyometric exercise to the traditional protocol was additive in this case, underlining the efficacy of the COMB protocol for improving isokinetic muscle strength in male athletes after ACL reconstruction.

Furthermore, the present study demonstrated that the COMB group outperformed the PLYO, ECC, and CON groups on psychological measurement kinesiophobia scores. A plausible explanation was that the COMB group imposed a more comprehensive stress of greater magnitude and improved psychological status by the way of increased confidence levels for the athlete. In addition, a closer inspection of the data showed that KOOS, IKDC, and TSK-CF questionnaire scores increased after the COMB-intervention, which agrees with reports of increased kinesiophobia at the time of return to sports after an injury [2, 8, 10].

To increase kinesiophobia, combined plyometric and eccentric exercise would seem appropriate. In fact, these training programs exposes patients to sports-related activities in a controlled setting [14] and graded exposure treatment is effective in athletes. However, graded exposure treatment focuses on activities that cause fear, and combined plyometric and eccentric exercises in this study were selected to gradually increase loads on the lower extremity.

Although novel, this study is not without limitations. First, the age range of subjects was narrow, which likely restricts extrapolation of our findings to other cohorts. Furthermore, a lack of nutritional control and of activities outside the training program should be considered as factors to potentially impact our outcomes. Moreover, our study results could be biased by learning effects. However, the participating individuals were accustomed to the applied test protocols (i.e., isokinetic stetting) because they participated in a familiarization session one week before the start of the study, allowing us to be confident that minimal-to-no impact of learning effects affected our results. In addition, we evaluated the effects of different RT exercises on isokinetic muscle strength and psychological statues in athletes following ACL reconstruction. However, we did not study the possible effects of these exercise modalities on other fitness tests that are relevant for a successful return to sport after the rehabilitation phase (i.e., change-of-direction speed). Future studies should, therefore, examine the effects of different rehabilitation training modalities on other fitness components, such as agility, speed, balance after anterior cruciate ligament reconstructions.

From a practitioner’s point of view, the most important outcome of this study is that combined eccentric and plyometric training is more effective to improve measures of isokinetic strength and psychological statues. Accordingly, combined eccentric and plyometric exercises should be preferred over single mode plyometric training during and following the rehabilitation period of training sessions in athletes.

Three sets of six maximal hurdle jumps over 20 cm combined with eccentric exercises appear to be sufficient to induce greater performance enhancements. While our study sample consisted of rather experienced youth athletes, findings of this study should be compared with those from novice and elite athletes. Accordingly, more research is needed in this area.

## Conclusion

Our results support the use of combined plyometric and eccentric training as a safe and effective strategy for psychological measurement kinesiophobia, functional knee assessment, and knee flexor and extensor isokinetic muscle strength performance of male athletes undergoing ACL reconstruction.

## Data Availability

The datasets generated and/or analysed during the current study are not publicly available. Upon request, the corresponding author will share the data set.

## Abbreviations

CON: control group
ECC: eccentric group
PLYO: plyometric group
COMB: combined group
F: Female
M: Male
L: Left
R: Right
T: Team sport
I: Individual sport
BMI: body mass index
TSK-CF: kinesiophobia
KOOS: knee injury and osteoarthritis outcome score
IKDC: international knee documentation committee 2000 questionnaire
PT: peak torque
TW: total work
R-HQ: ratio quadriceps/hamstring
R-TW: ratio of total work
